# Medial Branch Blocks for Diagnosis of Facet Joint Pain Etiology and Use in Chronic Pain Litigation

**DOI:** 10.3390/ijerph17217932

**Published:** 2020-10-29

**Authors:** Gordon E. Lawson, Paul S. Nolet, Adam R. Little, Anit Bhattacharyya, Vivian Wang, C. Adam Lawson, Gordon D. Ko

**Affiliations:** 1 Canadian Memorial Chiropractic College, Toronto, ON M2H 3J1, Canada; glawson@cmcc.ca (G.E.L.); vwang@cmcc.ca (V.W.); 2 Department of Graduate Education and Research, Canadian Memorial Chiropractic College, Faculty of Health, Medicine and Life Sciences, Maastricht University, Universiteitssingel 40, 6229 ER Maastricht, The Netherlands; p.nolet@maastrichtuniversity.nl; 3 Oatley Vigmond LLP, Barrie, ON L4M 6C1, Canada; alittle@oatleyvigmond.com; 4 Ontario Veterinary College, University of Guelph, Guelph, ON N1G 2 W1, Canada; anit@uoguelph.ca; 5 Shibley Righton LLP, Toronto, ON M5H 3E5, Canada; 6 Department of Medicine, Division of Physical Medicine and Rehabilitation, University of Toronto, Sunnybrook Health Sciences Centre, Toronto, ON M4N 3M5, Canada; gordon.ko@sunnybrook.ca

**Keywords:** forensic medicine, neck pain, nerve block, whiplash, zygapophyseal joint, medial branch blocks, diagnostic facet joint blocks, facet joint

## Abstract

A commonly disputed medicolegal issue is the documentation of the location, degree, and anatomical source of an injured plaintiff’s ongoing pain, particularly when the painful region is in or near the spine, and when the symptoms have arisen as result of a relatively low speed traffic crash. The purpose of our paper is to provide health and legal practitioners with strategies to identify the source of cervical pain and to aid triers of fact (decision makers) in reaching better informed conclusions. We review the medical evidence for the applications and reliability of cervical medial branch nerve blocks as an indication of painful spinal facets. We also present legal precedents for the legal admissibility of the results of such diagnostic testing as evidence of chronic spine pain after a traffic crash. Part of the reason for the dispute is the subjective nature of pain, and the fact that medical documentation of pain complaints relies primarily on the history given by the patient. A condition that can be documented objectively is chronic cervical spine facet joint pain, as demonstrated by medial branch block (injection). The diagnostic accuracy of medial branch blocks has been extensively described in the scientific medical literature, and evidence of facet blocks to objectively document chronic post-traumatic neck pain has been accepted as scientifically reliable in courts and tribunals in the USA, Canada and the United Kingdom. We conclude that there is convincing scientific medical evidence that the results of cervical facet blocks provide reliable objective evidence of chronic post-traumatic spine pain, suitable for presentation to an adjudicative decision maker.

## 1. Introduction

**Figure 1:**
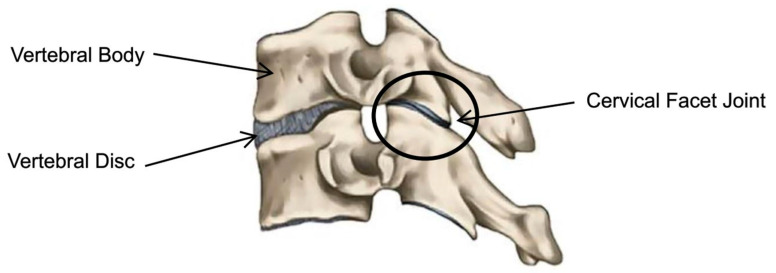
Cervical facet joint schematic.

Neck pain (NP) is a significant health problem globally, ranking fourth in terms of years lived with disability [1]. It also ranks 21st out of 291 diseases for the total burden of health using the disability adjusted life years (DALY) scale [2]. NP can be a chronic or recurrent condition that can impact health-related quality of life [3,4]. NP is common after an injury in a motor vehicle collision (MVC) with 86% of individuals reporting neck pain, and with the pain still occurring in 50% of those individuals a year later [5,6]. In a systematic review and meta-analysis of six studies with individuals who had sustained a neck injury in a MVC, those with neck injuries were at a greater risk of future neck pain when compared to those without a neck injury (RR = 2.3 [95% CI 1.8–3.1]) [7]. Cervical pain can have multiple etiologies, including cervical dystonia, instability, protrusion, and ligamentous damage. There are specific diagnostic protocols which would assist in identifying the sources of pain including flexion, extension, plain film x-ray, and MRI. The cervical facet joints (also known as zygapophysial joints) are a common anatomical source of pain in the post-MVC NP population [8]. Medial branch blocks (MBBs) are the best diagnostic test for identifying cervical facet etiological pain. There are two facet joints located posteriorly between each cervical vertebra of the C2-7 levels of the spine, each with their own joint capsule (Figure 1). The facet joint capsule is innervated by the medial branches of the dorsal primary ramus which transmit pain signals to the dorsal horn of the spinal cord from the facet joints of the same level of the spine [8].

The facet capsules of the neck can be strained in elongation, or synovial folds can be pinched during compression, both of which are mechanisms of injury that occur during whiplash trauma in an MVC [8]. Animal studies have shown that tear-causing strain to the facet capsule can lead to persistent firing of the afferent nerves that innervate the facet capsule, even without any evidence of tissue failure [8,9]. Conversely, there is evidence that greater injury to the facet joint capsule causing tissue failure need not produce persistent firing of the afferent nerves and thus does not necessarily produce persistent pain [9]. The cascade of events in sub-tissue failure injury within the facet capsule of rats can increase activity in the afferent nerves leading to changes in the dorsal horn of the spinal cord, consistent with central sensitization and persistent pain [10,11]. In addition, afferent nerves innervate more than one facet joint and hence it is important to investigate multiple joints to identify the correct source of the pain [12]. Given the basic-science evidence that injury to cervical facet joint can become an ongoing source of pain, it is important to be able to objectively diagnose pain emanating from the cervical facet joints in individuals who are suffering from persistent neck pain post-MVC [9].

Individuals can end up in litigation after an MVC due to the prolonged symptoms associated with neck injury. Litigation of neck injuries in MVC is costly to both the courts, clients, insurers, lawyers and medical professionals. In a medicolegal setting it is important to establish general causation between an injury and neck pain, and a specific causation with the individual, where a clinician determines a diagnosis which can then help determine a specific cause [13]. It is therefore important to be able to establish neck pain from facet joint injury in individuals with tests that are both reliable and valid as these can provide evidence of injury for the courts in order for them to be able to make a determination on causality. The purpose of this pragmatic review was to examine the medical literature on the use of tests to establish cervical facet joint pain after neck injury in an MVC, and to help inform the courts on causation.

## 2. Methodology

### 2.1. Literature Search Parameters

A clinical literature search strategy was developed in collaboration with a health sciences librarian, a copy of which is available on request from the authors. A keyword search and review of several international legal research services was conducted.

### 2.2. Search Strategy

An extensive search of the clinical literature was conducted in PubMed. Search terms consisted of subject headings and free text words relevant to facet joints, including the following terms: facet joint, zygapophyseal joint, nerve block, intra-articular injections, spinal injections, diagnosis, facet pain, prognosis. Publications were restricted to the English language and databases searched from inception to October 2018. Additionally, articles were selected by hand-searching reference lists to identify studies not fully indexed in electronic databases.

A similar search strategy was employed to search cases and legislation in several legal research services (WestLaw, QuickLaw, CanLII, and BAILII) using variants of terms such as “medial branch block”, “nerve block”, “diagnostic facet joint blocks”, “facet joint”, and “zygapophyseal”. Publications were restricted to the English language and databases searched from inception to September 2019.

## 3. Evaluating the Injured from a Medical Perspective

### 3.1. Clinical Presentation

**Figure 2:**
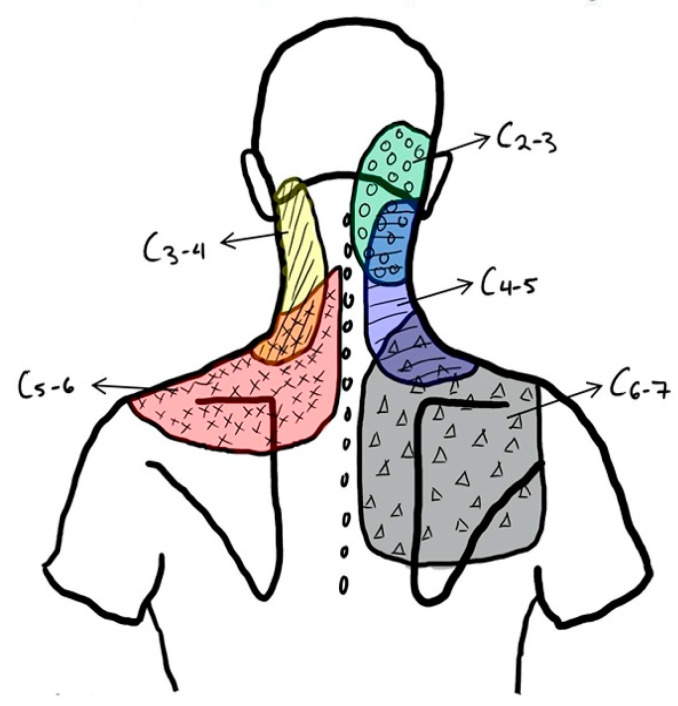
Cervical facet mediated pain radiation schematic [14].

The typical clinical presentation of an individual suffering from cervical facet mediated pain is generally unilateral neck pain, as well as pain on cervical rotation and extension [1]. In certain cases, pain radiation can occur segmentally and does so in specific patterns, not extending past the level of the shoulder (Figure 2) [14,15]. It is important to note that pain radiation can be produced segmentally by both the intervertebral disk and the zygapophyseal joint itself, which makes pain radiation patterns alone an insufficient tool for isolating the structure causing pain [2].

### 3.2. Clinical Examination

A thorough physical examination consists of range of motion, palpation, joint play, orthopedic testing and neurologic examination. Assessing the range of motion of the cervical spine can be a useful tool in assessing function and should be performed as part of a clinical examination. Patients nearly always self-report the range of motion increases when the MBB test is positive [16]. The reliability of manual examination in the diagnosis of cervical facet joint pain syndromes was recorded to be both highly sensitive (able to correctly identify those with the pain syndrome) and specific (able to correctly identify those without the pain syndrome) [17]. The diagnostic criteria for facet joint pain syndrome included unusual resistance to passive joint movement, abnormal end-feel, and reproduction of pain via passive movements [17]. The study conducted by Jull et al. used single diagnostic blocks as the reference standard. Multiple studies have now demonstrated that these single blocks are unreliable [18,19,20]. This study was limited in that it only used one therapist. A follow up study was completed in 2007 on a larger sample size by King et al., who used a comparative facet joint block criterion standard which has been validated as a diagnostic method [19,20,21,22,23,24,25,26]. It concluded that a physical examination of the cervical spine for the purpose of diagnosing of cervical facet pain lacked validity (the ability of a test to correctly diagnose those with cervical facet pain and those without) [27].

Mechanical pressure-pain threshold, also known as tenderness on palpation, is a component of the physical examination that has been evaluated and is not diagnostic for finding the specific cervical facet joints causing the symptoms [28]. It was found that tenderness was not restricted to the affected site but rather found to be more widespread [28].

There are not many clinical tests that can diagnose cervical facet joint pain syndrome. One of the most common is Kemp’s Test (also known as the maximal cervical facet compression test and the quadrant test) [29]. This test rotates the neck into extension and applies pressure downwards to compress the facet joints of the cervical spine [29]. Reproduction of pain is considered a positive test result, indicating that the facet joint is responsible for the subject’s pain [29]. In a systematic review of five articles addressing the validity of the Kemp’s Test in diagnosing facet joint pain, it was found that the test’s sensitivity and specificity was low [29]. Therefore, the Kemp’s Test on its own does not yield a meaningful result; however, when put into context with the clinical picture, it may have more use in ruling out facet joints as the source of pain [29]. Other provocative tests employed in the physical evaluation of the cervical spine were evaluated in a systematic review performed by Malanga et al. in 2003 [30]. It found fair to excellent reliability scores (based on kappa values), and generally high specificity and low sensitivity scores for Spurling’s Neck Compression Test, Shoulder Abduction Sign and Neck Distraction Test [30]. These are known to evaluate the presence of nerve root compression as a result of cervical disk pathologies (including degeneration, disk bulges, and herniation) which can impinge upon the nerve [30]. Although these are not targeted towards the diagnosis of cervical facet pain syndromes, they do help to rule out the possibility of a disc pathology, which we know can mimic the pain radiation of cervical facet joints.

Manual examination alone for tenderness of the C2–3 and C5–6 level of the cervical spine compared to a comparative medial branch block had a pooled sensitivity of 89% (95% CI 82–96%) and specificity of 47% (95% CI 37–57%) [27]. There is a lack of validity in the use of manual examination alone to diagnose facet joint pain [27].

In two more recent systematic reviews there was some preliminary evidence for the use of the extension-rotation (ER) test to rule out pain from cervical facet joints [31,32]. The ER test had adequate reliability in both comparing multiple measurements within the same examiner (intra-examiner reliability; kappa > 0.9) as well comparing measurements between different examiners (inter-examiner reliability; kappa > 0.7) [33]. The ER test also had adequate validity with an ability to correctly identify those with the pain (PPV) of 82.7% (95% CI 70.3–90.6%) and an ability to correctly identify those without the pain (NPV) of 58.9% (95% CI 70.3–90.6%) [34]. For both these tests, the ER test was compared to comparative MBBs as the reference standard. When palpation for segmental tenderness was added to the ER test there was an increase in the ability to correctly identify those without the pain to 83.4% (95% CI 73.4–90.3%) [34]. This leaves the ER test combined with additional palpation as a reliable and valid method to detect facet joint pain.

There is encouraging but variable evidence for the use of physical examination in diagnosing pain from the cervical facet joints, even though there is a strong importance placed on completing a thorough physical evaluation when faced with patient expectations. Whether clinicians meet these expectations may affect the satisfaction and recovery of the patients themselves [35].

Completing a comprehensive physical exam is essential in the practice of medicine. Whether or not a clinician meets the patient’s expectation of a thorough physical exam may affect the satisfaction and recovery of the patient [35]. However, it must be recognized that there are challenges associated with certain tests because of the variability of their sensitivity and specificity. The physical exam can be used as a screening tool for patients who would benefit from the more robust testing of medial branch blocks (MBB). The term MBB can also be referred to as medial branch nerve block (MBNB) or a diagnostic facet joint block (DFJB). The terms are often used interchangeably. The information provided by these physical tests will be important for the recommendation of clinical management in medical legal decisions.

### 3.3. Diagnostic Imaging

There are many different imaging methods available to examine the anatomy of the spine. These include but are not limited to plain radiography, magnetic resonance imaging (MRI), computed tomography (CT), and radionuclide bone scans. Each test has specific strengths and weaknesses; however, most have limited and less than ideal power to identify facet joint pain.

Plain radiography is usually the first imaging tool used in evaluating neck pain. This is because it provides a through visualization of the skeletal anatomy which offers potential in evaluating bone changes and joint space changes, major facet joint changes in particular [36]. However, there is only a weak association between neck pain and radiographic evidence of spinal degeneration [37].

MRI is best at visualizing soft tissue structures and cannot visualize cortical bone. Therefore, it is not the best method of evaluating the cervical facet joints which are primarily made up of cortical bone [36].

Computed Tomography (CT) on the other hand is a great imaging tool for evaluating cortical bone and therefore a better method for observing the facets [36]. Despite this, it has been shown in other articles that CT images are not effective in diagnosing pain that originates from the cervical facet joints [38,39].

A promising imaging tool in the diagnosis of facet joint pain is radionuclide bone scan, also known as bone scintigraphy. Radionuclide bone scans are able to detect alterations in bone earlier than plain radiographs can and are also able to examine almost the entire skeleton at one time [36]. Therefore, it is able to indicate the level in which issues occur [36]. It has been found that radionuclide bone scans with single-photon emission computed tomography (SPECT) can help identify patients who may benefit from facet joint injections and may help in selecting patients that should undergo the more invasive facet injection procedures [40,41]. More recently, it has been discovered that bone SPECT-CT scans are more reliable than bone SPECT in identifying facet disease; however, it is still only moderately specific and sensitive when compared to diagnostic nerve blocks in diagnosing symptomatic facets [42,43]. These tests are not widely used at this time due to radiation concerns.

## 4. Cervical Medial Branch Blocks for Diagnostic Facet Joint Pain Etiology

The highest standard utilized in MBB testing is fluoroscopy. In clinical settings when fluoroscopy is not available, diagnostic ultrasound is sometimes used. This is an improvement over blinded testing where no diagnostic assistance is available but will not provide the same degree of certitude in identifying the location of the joint and therefore the medial branch of the dorsal ramus. Diagnostic ultrasound is useful for improving accuracy in locating blood vessels and ligamentous tissue (e.g., iliolumbar, sacroiliac posterior interosseous) injections, but the focus of this paper is specifically on spinal facet joints [44]. Ultrasound requires selection of the appropriate patient (healthy tissues, not obese, and able to achieve appropriate posture). The process requires greater time and skill on the part of the assessor.

Fluoroscopy is the preferred method utilized by clinicians performing MBBs. It employs a sterile technique using spinal needles inserted towards the cervical articular pillars via a lateral approach [19]. The target is patient specific and consists of unilateral or bilateral medial branches of the cervical dorsal rami that supply the facet joints of C3–C7 and the third occipital nerve which supplies the C2–C3 facet joint [45]. There are three major methods: single facet nerve blocks, placebo-controlled facet nerve blocks, and comparative controlled facet nerve blocks.

Single cervical facet blocks employ the use of a single injection of a local anesthetic. A positive response would consist of a definite or complete relief of pain after the injection [19]. This has been shown in the literature to have a high false positive rate of 27% to 45% which makes the method an invalid tool in diagnosing cervical facet pain [18,19]. This makes single cervical facet blocks both clinically and legally irrelevant. The other two methods are forms of controlled nerve block which are needed to make diagnostic nerve blocks a valid diagnostic tool. In fact, blinded controlled diagnostic facet joint blocks are the best available method to diagnose chronic facet joint pain and have been claimed to be the current gold standard [18,46].

Placebo-controlled nerve blocks employ the use of three injections, one of lignocaine, one of bupivacaine, and one of normal saline. Although there are benefits in the validity of placebo-controlled nerve blocks, it has some weaknesses in comparison to comparative nerve blocks. These are additional time consumption, additional resource consumption, greater risk with number of injections, and possible ethical dilemma in private practice [22].

Comparative diagnostic nerve blocks have been shown in many studies to be specific, valid, reliable, reproducible, safe and utilizable in the diagnosis of cervical facet mediated pain [19,20,22,23,24,25,26]. The methodology is similar to previously described blocks, but instead uses a total of two injections, one local anesthetic with short lasting relief (lignocaine) and another with long lasting relief (bupivacaine), and has six possible responses [19,45].

Those experiencing complete relief are placed into two major possible response groups, concordant and discordant (Table 1). Both concordant and discordant response categories signify complete relief of pain; however, concordant responses signify the correct duration for the local anesthetic, where discordant responses signify the incorrect duration for the local anesthetic [45]. A concordant positive response has been shown to have a high specificity of 88% but a low sensitivity of 54% when compared to a placebo-controlled nerve block [22]. This means that all concordant responses are likely to be true positives for facet joint pain. However, not all patients with facet joint pain are detected [45]. When the diagnostic outcome was expanded to all patients with reproducible pain relief regardless of duration, the specificity was reduced to 65% and the sensitivity increased to 100% [22]. In either case, it has been noted by that, as prevalence of facet mediated pain rises, the diagnostic confidence (or positive predictive value) increases accordingly [45]. Given that the prevalence rate of cervical facet mediated pain ranges between 40–68%, a 60% prevalence rate would place the diagnostic confidence at 87% [45,47].

A best evidence systematic appraisal by Boswell et al. (2015) reported level two evidence (multiple low to moderate quality accuracy studies) for the use of facet blocks in the diagnosis of facet joint pain. Over the eleven diagnostic accuracy studies published they found a prevalence of facet joint pain between 36–67%, when using 80% as the pain relief criteria. There was a high false positive rate ranging from 27–63%. A more recent study of 45 subjects with chronic whiplash associated disorder, using 80% relief with medial branch blocks and a double-blind technique (with both placebo saline and bupivacaine), yielded 29% true positive responders, 60% non-responders and 11% placebo responders [22].

Cervical diagnostic facet blocks have had detractors who have questioned the reliability and validity of the test for diagnosing cervical facet joints as the source of the patient’s pain [48,49,50]. A MBB for cervical facet pain has not been validated against a gold standard in order to be able to establish facet joint pain as the primary cause of neck pain in patients [49]. Further, there is no established gold standard available to test the MBB against in order to validate the test. In order to establish the validity of the MBB in the absence of a gold standard, the results of the index test would need to be examined for the association with relevant clinical characteristics [51]. Future research on the use of MBB for diagnosis of cervical facet joint pain should examine associations between the test and future outcome in prognosis studies, or healthcare outcomes in randomized controlled treatment studies. This type of research looks at the meaningfulness of the Cervical MBB instead of just examining the accuracy of the test [51]. Validation studies can help explain the extent to which the MBB test fits our understanding of facet joint pain, the probable causes, clinical course, and results of treatment interventions, and thus help direct its use in a medical and legal setting [52].

## 5. Evaluating an Injured Person from a Litigation Perspective

One of the key aspects to successful chronic pain litigation is credibility—how believable your client and their claim for injuries is. For most personal injury lawyers, the initial interview and document collection stages are the first opportunity to evaluate a client’s injuries, level of disability, and overall credibility. However, as the case progresses, depositions/examinations for discovery and medico-legal assessments are also critical in evaluating the strength of a client’s case. A client’s credibility is especially important in chronic pain claims caused by whiplash injuries because the symptoms are usually entirely subjective in nature. If the client lacks credibility, their subjective complaints are less likely to be accepted by the trier of fact (the tribunal, judge, or jury).

Where chronic pain is the chief complaint, objective evidence can bolster and help corroborate the client’s report of their seemingly subjective symptoms in the courtroom. In reviewing Canadian case law, diagnostic facet block injections have provided objective evidence and appear to be an underutilized but accepted tool for assessment of back or neck pain. As such, there should be consideration given to retaining a specialist in pain management to perform medial branch block injections in cases where subjective symptoms of neck pain are primarily at issue and there is no obvious objective initial injury, such as a fracture.

### Using Facet Testing in a Legal Case

In Canadian courts and insurance tribunals, diagnostic facet block testing can better establish the causation of pain and has been accepted as objective evidence in support of a diagnosis of facet injury and chronic pain. Currently its use as an evidentiary tool is not yet widespread, though it has been relied upon by tribunals and courts in the United Kingdom and United States of America [53,54,55,56,57].

In Canada and elsewhere, precedential case law has established that the common law understands that chronic pain is not always supported by objective findings at the site of injury under the current medical techniques. In *Martin v. Nova Scotia (Workers’ Compensation Board)*, the Nova Scotia workers’ compensation scheme was challenged by two workers who suffered from chronic pain, which had been excluded as a compensable injury by the legislature [58]. The Supreme Court of Canada decided that, despite the lack of objective findings, there was no doubt that these chronic pain patients were suffering and in distress, and that the disability they experienced was real [58]. The workers’ compensation legislation was found to be discriminatory against those suffering from chronic pain, and thus unconstitutional. *Martin v. Nova Scotia* was considered to be a major step forward for chronic pain in Canadian courts.

However, despite the Supreme Court’s findings in *Martin v. Nova Scotia,* and a confirmation that MVC-related chronic pain is compensable even without objective findings, the desire to prove the existence of subjective pain using objective evidence in the courtroom continues [59,60]. This is because the burden of proof regarding a claim falls entirely on the injured person (the plaintiff). Objective evidence can help persuade the court in the plaintiff’s favor in a chronic pain case, where injuries are usually based entirely on subjective complaints. In these cases, the credibility of the plaintiff often becomes the central issue at trial. A plaintiff whose complaints of chronic pain are supported by objective evidence from diagnostic tests is more likely to succeed at trial.

An illustrative example of a subjective chronic pain claim bolstered by MBB/MBNB/DFJB testing is *McDonald v. Kwan* [61]. The plaintiff brought an action for damages arising from a rear-end motor vehicle collision resulting in persistent headaches and neck pain. At trial, the plaintiff’s credibility was forcefully challenged. The defense asked the court to reject the plaintiff’s evidence as unreliable, and to find that his complaints were primarily a product of conscious symptom magnification motivated by a desire for financial gain. The orthopedic surgeon retained by the defense testified there was nothing physically wrong with the plaintiff, because neither the radiological investigations nor his examination revealed any objective findings correlated to his ongoing subjective complaints. The plaintiff’s treating physiatrist (one of the authors, Dr. Gordon Ko) testified that he had arranged for diagnostic facet block testing approximately a year and a half after the collision. The results were positive for injury to the cervical facet joints, and surgical treatment was recommended, although never conducted. The defense orthopedic surgeon was skeptical of the reliability of diagnostic facet block testing and the objectivity of its results, contending that the subjective element had not been adequately eliminated from the testing process and that the mechanics of the test left room for inaccuracy. In reaching a decision in favor of the plaintiff, the trial judge stated:

It is neither within the scope of this action nor possible on the evidence before the court to make a definitive finding as to the efficacy of facet joint blocks in identifying injury to those joints. What I must decide is whether the evidence in support of the facet blocks, having objectively identified Mr. McDonald’s cervical facet joints as a source of his pain, is more compelling than the countervailing evidence. In my view, it is. In reaching this conclusion, I am influenced by the fact that the doctors who support the efficacy of facet blocks have expertise in the diagnosis and treatment of headaches and neck pain. This is in contrast to the orthopaedic surgery area of specialisation of Dr. Bushuk [the defence expert].

I accept that the diagnostic study conducted by Dr. Shapero in December 2001 provides objective evidence confirming the injury to Mr. McDonald’s cervical facet joints and that they were a source of his chronic pain [61].

Similarly, in *Cobb v. Long Estate* [62], the plaintiff’s subjective experience of chronic pain following a frontal motor vehicle collision was questioned by the defense. They alleged malingering and a lack of objective evidence of injury. The physiatrist retained by the plaintiff (Dr. Gordon Ko) testified that diagnostic facet block testing provided objective evidence of injury to the facet joints. The defense’s orthopedic surgeon argued against the analysis by suggesting that injections of saline solution might produce the same results. Based on the totality of the evidence, the trial judge concluded that the plaintiff had suffered a permanent and serious impairment of an important bodily function and was therefore entitled to compensation.

Courts in British Columbia have repeatedly accepted MBBs as legitimate costs of future care and important treatment and as diagnostic tools [63,64,65]. In the British Columbia case of *Vienneau v. Dhaliwal* [66], a case based entirely on subjective symptoms and with a plaintiff found to have a “lack of credibility” [66], the trial judge nonetheless accepted that the plaintiff’s symptoms were caused by facet joint pathology from a MVC because of MBB testing, regardless of the plaintiffs credibility. After accepting testimony from both the plaintiff and defense expert who opined that medial branch blocks were “the gold standard” for diagnosing the facet joints, and had provided temporary pain relief, he stated:

If the medial branch block procedure is the gold standard for diagnosing facet joint injury, and [a physician] successfully performed that procedure on the plaintiff, then I see no reason not to conclude that the plaintiff’s symptoms are caused by cervical facet joint pathology [66].

In the arena of workers’ compensation claims, evidence of diagnostic facet block testing has been accepted and relied upon several times by the Ontario Workplace Safety and Insurance Appeals Tribunal [67,68,69,70,71,72,73,74,75]. For example, in *Decision No. 2174/04* [76], the Tribunal held that medical evidence (including diagnostic facet block testing) supported a finding that the worker had sustained a chronic facet joint problem and was entitled to benefits. Similarly, in *Decision No. 2460/10* [72], evidence of a negative diagnostic facet block test was used to exclude a diagnosis of facet joint pain syndrome.

Diagnostic nerve block testing has also been accepted as objective evidence of injury by the Financial Services Commission of Ontario, in adjudicating claims for statutory accident benefits. In *Lazareva v. Royal Insurance Co. of Canada* [77], the insured person underwent MBB testing, the results of which were positive. When physicians retained by the insurer disputed the results of the testing, the insured retained Dr. Nikolai Bogduk (a leading authority on the protocol for diagnostic facet joint blocks/medial branch block administration). Dr. Bogduk provided evidence that the insured had a genuine source of pain in her facet joints. The Commission Arbitrator concluded:

I accept Dr. Bogduk’s opinion that the diagnostic nerve blocks performed on Ms. Lazareva constitute an objective finding of genuine cervicogenic pain. Dr. Bogduk may have shared Dr. Watson’s misgivings about the way in which Dr. Shapero conducted the blocks but he did not share Dr. Watson’s opinion about the reliability of the results obtained [77].

Overall, the results of medial branch nerve block testing have been accepted as objective evidence of injury by judges and tribunals in Canada, albeit infrequently. As noted previously, the primary benefit of such test results is the addition of objective evidence to an otherwise entirely subjective claim.

## 6. Conclusions

A review of the medical and legal literature for MBB/MBNB/DFJB utilization demonstrates a sound basis for its utilization in medicine. It is an accepted diagnostic procedure that is utilized within the medical community. The results of MBBs have been used by tribunals and courts of law to assist the trier of fact in establishing the physical etiology of pain and determining the effect of physical trauma on function. It thereby assists in determining the causation of the injury, mitigation of its effects, and promotes resolution by providing more information for assessment.

As with all elements of science, the basis of information is founded on probability. There is not mathematical certitude of the conclusions, but the foundation of the MBB test is on a sound scientific footing. With growing awareness, understanding, and reliance on MBBs in courts of law this testing should become more widespread and used with greater frequency for better adjudicative decision-making.

The usage of MBBs in clinical practice is significant. A more specific and accurate diagnosis will allow the practitioner to focus on the optimal therapeutic intervention: platelet rich plasma or rhizotomy if the testing is positive; and conservative intervention or further investigations if the testing is negative.

The focus on this paper is the use of MBBs in medical-legal settings. Positive MBBs can strengthen the evidence provided to courts of law and tribunals and state, with strong support, that there is a physical source of the pain as a result of the injury. MBBs should be used as a standard protocol in preparing legal cases involving cervical pain as a result of trauma.

## Figures and Tables

**Table 1:** Comparative diagnostic nerve block response categories. Adapted from The utility of comparative local anesthetic blocks versus placebo- controlled blocks for the diagnosis of cervical zygapophysial joint pain. [22].

Response Group	Definition
Concordant	Longer pain relief with bupivacaine <7 h of pain relief with lignocaine <24 h with bupivacaine
Concordant prolonged	Longer pain relief with bupivacaine >7 h of pain relief with lignocaine >24 h of pain relief with bupivacaine
Discordant	Longer pain relief with lignocaine, <7 h of pain relief with lignocaine <24 h of pain relief with bupivacaine
Discordant prolonged	Longer pain relief with lignocaine >7 h of pain relief with lignocaine >24 h of pain relief with bupivacaine
Discrepant	Pain relief with only one of the local anesthetics
Negative	No relief at any cervical level from either local anesthetic
